# Supplementary material for: Effects of an obesogenic diet on the oviduct depend on the duration of feeding
Source: PLoS One. 2022 Sep 29;17(9):e0275379. doi: 10.1371/journal.pone.0275379 (PMC9522283; doi:10.1371/journal.pone.0275379)
Supplement: S1 Table — (PDF) [file pone.0275379.s001.pdf]

S1 Table. Functions and full names of cytokines measured in the serum

Cytokine	Full name	Function
GM-CSF	Granulocyte macrophage colony-stimulating factor	Monomeric glycoprotein that functions as a cytokine. Stimulates stem cells to produce granulocytes (neutrophils, eosinophils and basophils) [1].
IFN-β	Interferon β	Type I interferon. Induces the transcription of genes encoding inflammatory cytokines and chemokines [2].
IL-1α	Interleukin 1α	Inflammatory cytokine that activates the inflammatory process [3]. Cytokine required for activating the innate immune response [4].
IFN-γ	Interferon γ	Type II interferon with an antiviral activity [5].
MCP-1	Monocyte chemoattractant protein-1	Chemokine that regulates the migration and infiltration of monocytes/macrophages [2].
TNF-α	Tumor necrosis factor α	Inflammatory cytokine produced by macrophages/monocytes during acute inflammation. Responsible for a diverse range of signalling events within cells, leading to necrosis or apoptosis [6]. Cytokine required for activating the innate immune response [4].
IL-17A	Interleukin 17A	Proinflammatory cytokine produced by activated T cells. Stimulates the expression of IL6 and COX-2 [7].
IL-23	Interleukin 23	Pro-inflammatory cytokine that stimulates the production of IL-17 [8].
IL-10	Interleukin 10	Anti-inflammatory cytokine that limits the production of proinflammatory cytokines and chemokines [9].
IL-6	Interleukin 6	Both a pro-inflammatory and an anti-inflammatory cytokine. Induces different acute phase proteins and IL-10 but has also inhibitory effects in TNF-α and IL-1 [10].
IL-27	Interleukin 27	Has both pro-inflammatory and anti-inflammatory properties. Considerable conflicting data exists about the role of IL-27 [11].
IL12p70	Interleukin 12p70	Pro-inflammatory cytokine. Induces IFN- γ and TNF-α production [12].
IL-1β	Interleukin 1β	Pro-inflammatory cytokine. Synergizes with TNF to produce IL-6. Synergize with IL-23 to induce IL-17 production [7].

1. Becher B, Tugues S, Greter M. GM-CSF: From Growth Factor to Central Mediator of Tissue Inflammation. Immunity. 2016;45(5):963-73.
2. GeneCards. [Available from: https://www.genecards.org/.
3. Di Paolo NC, Shayakhmetov DM. Interleukin 1alpha and the inflammatory process. Nat Immunol. 2016;17(8):906-13.
4. Ott LW, Resing KA, Sizemore AW, Heyen JW, Cocklin RR, Pedrick NM, et al. Tumor Necrosis Factor-alpha- and interleukin-1-induced cellular responses: coupling proteomic and genomic information. J Proteome Res. 2007;6(6):2176-85.
5. Tau G, Rothman P. Biologic functions of the IFN-gamma receptors. Allergy. 1999;54(12):1233-51.
6. Idriss HT, Naismith JH. TNF alpha and the TNF receptor superfamily: structure-function relationship(s). Microsc Res Tech. 2000;50(3):184-95.
7. Zenobia C, Hajishengallis G. Basic biology and role of interleukin-17 in immunity and inflammation. Periodontol 2000. 2015;69(1):142-59.
8. Tang C, Chen S, Qian H, Huang W. Interleukin-23: as a drug target for autoimmune inflammatory diseases. Immunology. 2012;135(2):112-24.
9. Couper KN, Blount DG, Riley EM. IL-10: the master regulator of immunity to infection. J Immunol. 2008;180(9):5771-7.
10. Tanaka T, Narazaki M, Kishimoto T. IL-6 in inflammation, immunity, and disease. Cold Spring Harb Perspect Biol. 2014;6(10):a016295.
11. Carl JW, Bai XF. IL27: its roles in the induction and inhibition of inflammation. Int J Clin Exp Pathol. 2008;1(2):117-23.
12. Gee K, Guzzo C, Che Mat NF, Ma W, Kumar A. The IL-12 family of cytokines in infection, inflammation and autoimmune disorders. Inflamm Allergy Drug Targets. 2009;8(1):40-52.
