# Supplementary material for: Effects of an obesogenic diet on the oviduct depend on the duration of feeding
Source: PLoS One. 2022 Sep 29;17(9):e0275379. doi: 10.1371/journal.pone.0275379 (PMC9522283; doi:10.1371/journal.pone.0275379)
Supplement: S2 Table — (PDF) [file pone.0275379.s002.pdf]

S2 Table. Functions and full names of genes of interest used for qPCR

Gene	Full name	Domain	Function
SOD2	Superoxide dismutase 2	Oxidative stress	Mitochondrial enzyme that helps maintain the redox balance by converting superoxide radicals to hydrogen peroxide [1].
NRF1	Nuclear respiratory factor 1	Oxidative stress	Binds to ARE (antioxidant response element) and regulates the expression of a number of genes involved in oxidative stress [2].
PRDX1	Peroxiredoxin-1	Oxidative stress	Antioxidant protein, constitute a potent defence system for maintaining redox balance by converting hydrogen peroxide to water [3].
PRDX3	Peroxiredoxin-3 / Thioredoxin-dependent peroxide reductase	Oxidative stress	Antioxidant protein, constitute a potent defence system for maintaining redox balance by converting hydrogen peroxide to water [3].
PRDX6	Peroxiredoxin-6	Oxidative stress	Antioxidant protein, constitute a potent defence system for maintaining redox balance by converting hydrogen peroxide to water [3].
NRF2	Nuclear factor erythroid 2-related factor 2	Oxidative stress	Binds to ARE (antioxidant response element) and regulates the expression of a number of genes involved in oxidative stress [2].
BiP (HSPA5)	Binding immunoglobulin protein (Heat Shock Protein Family A Member 5)	Endoplasmic reticulum stress	Involved in maintaining proper protein folding and assembly in the ER [4].
ATF4	Activating transcription factor 4	Endoplasmic reticulum stress	Coordinates the response to oxidative stress and ER-stress by promoting expression of genes linked to resistance to oxidative stress [5].
HSPE1	Heat shock protein family E member 1	Mitochondrial stress	Assists folding of proteins in the mitochondrial matrix space [6].
HSPD1	Heat shock protein family D member 1	Mitochondrial stress	Responsible for (re)-folding of nuclear-encoded proteins that are imported into the mitochondria [7].
HSPA8	Heat shock protein family A member 8	Protein folding (chaperons)	Plays a role in protein quality control, ensuring the correct folding and re-folding of selected proteins [8].
IL-1β	Interleukin-1beta	Inflammation	Inflammatory cytokine [9].

1. Ashtekar A, Huk D, Magner A, La Perle KMD, Boucai L, Kirschner LS. Alterations in Sod2-Induced Oxidative Stress Affect Endocrine Cancer Progression. J Clin Endocrinol Metab. 2018;103(11):4135-45.
2. Biswas M, Chan JY. Role of Nrf1 in antioxidant response element-mediated gene expression and beyond. Toxicol Appl Pharmacol. 2010;244(1):16-20.
3. Jeong SJ, Kim S, Park JG, Jung IH, Lee MN, Jeon S, et al. Prdx1 (peroxiredoxin 1) deficiency reduces cholesterol efflux via impaired macrophage lipophagic flux. Autophagy. 2018;14(1):120-33.
4. Wang J, Pareja KA, Kaiser CA, Sevier CS. Redox signaling via the molecular chaperone BiP protects cells against endoplasmic reticulum-derived oxidative stress. Elife. 2014;3:e03496.
5. Lange PS, Chavez JC, Pinto JT, Coppola G, Sun CW, Townes TM, et al. ATF4 is an oxidative stress-inducible, prodeath transcription factor in neurons in vitro and in vivo. J Exp Med. 2008;205(5):1227-42.
6. Bie AS, Fernandez-Guerra P, Birkler RI, Nisemblat S, Pelnena D, Lu X, et al. Effects of a Mutation in the HSPE1 Gene Encoding the Mitochondrial Co-chaperonin HSP10 and Its Potential Association with a Neurological and Developmental Disorder. Front Mol Biosci. 2016;3:65.
7. Klebl DP, Feasey MC, Hesketh EL, Ranson NA, Wurdak H, Sobott F, et al. Cryo-EM structure of human mitochondrial HSPD1. iScience. 2021;24(1):102022.
8. Bonam SR, Ruff M, Muller S. HSPA8/HSC70 in Immune Disorders: A Molecular Rheostat that Adjusts Chaperone-Mediated Autophagy Substrates. Cells. 2019;8(8).

9. Madan JC, Davis JM, Craig WY, Collins M, Allan W, Quinn R, et al. Maternal obesity and markers of inflammation in pregnancy. Cytokine. 2009;47(1):61-4.
