# Supplementary material for: Effects of an obesogenic diet on the oviduct depend on the duration of feeding
Source: PLoS One. 2022 Sep 29;17(9):e0275379. doi: 10.1371/journal.pone.0275379 (PMC9522283; doi:10.1371/journal.pone.0275379)
Supplement: S3 Table — (PDF) [file pone.0275379.s003.pdf]

S3 Table. Pearson correlation Swiss mice

		Weight	Cholesterol	*BiP*	*PRDX1*	*HSPE1*	*SOD2*	*PRDX6*	*PRDX3*	*NRF2*	*HSPD1*	*HSPA8*	*ATF4*	*NRF1*	*IL-1 β*
**Weight**	Pearson Correlation	1													
	Sig. (2-tailed)														
**Cholesterol**	Pearson Correlation	.322**	1												
	Sig. (2-tailed)	0,008													
BiP	Pearson Correlation	0,205	.330*	1											
	Sig. (2-tailed)	0,193	0,035												
PRDX1	Pearson Correlation	-0,027	.361*	0,117	1										
	Sig. (2-tailed)	0,864	0,02	0,46											
HSPE1	Pearson Correlation	0,01	0,052	.597**	-0,135	1									
	Sig. (2-tailed)	0,952	0,748	0	0,393										
SOD2	Pearson Correlation	0,163	.414**	0,289	.683**	0,033	1								
	Sig. (2-tailed)	0,302	0,007	0,063	0	0,835									
PRDX6	Pearson Correlation	.563**	.359*	0,186	.330*	0,07	.349*	1							
	Sig. (2-tailed)	0	0,025	0,25	0,038	0,669	0,027								
PRDX3	Pearson Correlation	-0,165	0,123	0,282	.551**	0,012	.526**	0,105	1						
	Sig. (2-tailed)	0,297	0,442	0,07	0	0,938	0	0,521							
NRF2	Pearson Correlation	-0,211	-0,216	-0,074	0,01	-0,122	0,028	-0,141	.325*	1					
	Sig. (2-tailed)	0,179	0,176	0,639	0,948	0,442	0,858	0,385	0,036						
HSPD1	Pearson Correlation	-0,243	-0,209	-0,032	0,195	-0,072	-0,014	-0,177	.628**	.580**	1				
	Sig. (2-tailed)	0,121	0,189	0,842	0,216	0,651	0,928	0,275	0	0					
HSPA8	Pearson Correlation	-0,258	-0,205	0,024	0,114	-0,025	0,138	-0,193	.565**	.924**	.750**	1			
	Sig. (2-tailed)	0,099	0,2	0,878	0,47	0,874	0,382	0,234	0	0	0				
ATF4	Pearson Correlation	-0,122	-0,251	-0,136	0,193	-0,218	0,099	-0,106	.560**	.720**	.832**	.795**	1		
	Sig. (2-tailed)	0,44	0,114	0,389	0,221	0,165	0,531	0,513	0	0	0	0			
NRF1	Pearson Correlation	-0,204	-0,199	-0,026	0,072	-0,135	-0,025	0,029	.448**	.918**	.801**	.916**	.842**	1	
	Sig. (2-tailed)	0,194	0,213	0,872	0,649	0,393	0,873	0,857	0,003	0	0	0	0		
IL-1 β	Pearson Correlation	.684**	.479*	0,07	0,381	-0,043	.540**	.492*	0,13	-0,17	-0,203	0,04	0,236	0,348	1
	Sig. (2-tailed)	0	0,015	0,733	0,055	0,833	0,004	0,011	0,525	0,407	0,32	0,844	0,247	0,081	

** Correlation is significant at the 0.01 level (2-tailed)

* Correlation is significant at the 0.05 level (2-tailed)
