# Supplementary material for: Effects of an obesogenic diet on the oviduct depend on the duration of feeding
Source: PLoS One. 2022 Sep 29;17(9):e0275379. doi: 10.1371/journal.pone.0275379 (PMC9522283; doi:10.1371/journal.pone.0275379)
Supplement: S4 Table — (PDF) [file pone.0275379.s004.pdf]

S4 Table. Pearson correlation B6 mice

		Weight	Cholesterol	*BiP*	*PRDX1*	*HSPE1*	*SOD2*	*PRDX6*	*PRDX3*	*NRF2*	*HSPD1*	*HSPA8*	*ATF4*	*NRF1*	*IL-1 β*
**Weight**	Pearson Correlation	1													
	Sig. (2-tailed)														
**Cholesterol**	Pearson Correlation	.536**	1												
	Sig. (2-tailed)	0													
BiP	Pearson Correlation	-0,047	0,22	1											
	Sig. (2-tailed)	0,766	0,162												
PRDX1	Pearson Correlation	-0,215	-0,16	0,04	1										
	Sig. (2-tailed)	0,171	0,312	0,802											
HSPE1	Pearson Correlation	-0,031	-0,099	-0,194	.409**	1									
	Sig. (2-tailed)	0,845	0,533	0,218	0,007										
SOD2	Pearson Correlation	-0,173	0,009	0,186	.443**	0,106	1								
	Sig. (2-tailed)	0,273	0,955	0,237	0,003	0,502									
PRDX6	Pearson Correlation	-0,045	0,005	0,103	.322*	0,052	0,208	1							
	Sig. (2-tailed)	0,779	0,974	0,515	0,037	0,743	0,187								
PRDX3	Pearson Correlation	-0,068	-0,105	0,085	.560**	0,032	.601**	.470**	1						
	Sig. (2-tailed)	0,669	0,509	0,59	0	0,841	0	0,002							
NRF2	Pearson Correlation	0,09	0,199	.478**	0,259	-0,079	0,118	.439**	.308*	1					
	Sig. (2-tailed)	0,571	0,206	0,001	0,097	0,618	0,455	0,004	0,047						
HSPD1	Pearson Correlation	-0,039	0,14	.462**	0,11	-0,045	0,027	.499**	0,118	.826**	1				
	Sig. (2-tailed)	0,806	0,377	0,002	0,487	0,776	0,864	0,001	0,455	0					
HSPA8	Pearson Correlation	-0,01	0,188	.649**	0,125	-0,038	-0,048	.417**	0,15	.772**	.840**	1			
	Sig. (2-tailed)	0,948	0,234	0	0,429	0,813	0,764	0,006	0,345	0	0				
ATF4	Pearson Correlation	-0,071	0,194	.665**	0,172	-0,248	0,221	.472**	0,226	.748**	.775**	.808**	1		
	Sig. (2-tailed)	0,655	0,219	0	0,276	0,113	0,161	0,002	0,151	0	0	0			
NRF1	Pearson Correlation	-0,069	0,056	.425**	-0,033	-0,104	0,122	.335*	0,09	.741**	.882**	.627**	.696**	1	
	Sig. (2-tailed)	0,664	0,727	0,005	0,837	0,513	0,44	0,03	0,569	0	0	0	0		
IL-1 β	Pearson Correlation	-0,175	-0,089	-0,003	.644**	0,109	0,307	0,133	.558**	.398*	0,19	0,093	0,169	0,174	1
	Sig. (2-tailed)	0,392	0,666	0,989	0	0,595	0,127	0,517	0,003	0,044	0,353	0,65	0,409	0,394	

** Correlation is significant at the 0.01 level (2-tailed)

* Correlation is significant at the 0.05 level (2-tailed)
