# Supplementary material for: Effects of an obesogenic diet on the oviduct depend on the duration of feeding
Source: PLoS One. 2022 Sep 29;17(9):e0275379. doi: 10.1371/journal.pone.0275379 (PMC9522283; doi:10.1371/journal.pone.0275379)
Supplement: Attachment — Submitted filename: 20220710 PLOS One Review comments.docx [file pone.0275379.s005.docx]

In this paper, Kerlijne and colleagues determined the effect of obesogenic diet on the oviduct of mice during different feed duration. They founded that HF/HS elevated ER-stress and oxidative stress genes in OECs after 3days and mitochondrial antioxidants were upregulated after one week. Serum inflammatory cytokines were increased during the late-phase. In general, the present manuscript is a superficial study with very limited data present. I have the following comments for the authors to address before further consideration.

1. All 6 figures across the manuscript can be combined to 2 or 3 figures. The schematic diagram is far away from representing as a dependent figure.
2. Was fasting preformed before sacrifice the mice?
3. All figures were present in low resolution.
4. Most of the data present in the study was from RT-PCR which will compromised the convincing of the results.
5. The inflammatory cytokines were detected in the serum. Does these cytokines were detected in the OECs?
